# Analysis of endophytic bacterial diversity of *Puerariae thomsonii* from different production areas and their correlation with secondary metabolites

**DOI:** 10.3389/fmicb.2025.1534308

**Published:** 2025-05-26

**Authors:** Yang Xu, Lihua Zeng, Zheng Peng, Nana Chang, Ye Wang, Lingling Zheng, Yan Ren, Hui Li, Tielin Wang

**Affiliations:** ^1^Jiangxi Province Key Laboratory of Sustainable Utilization of Traditional Chinese Medicine Resources, Institute of Traditional Chinese Medicine Health Industry, China Academy of Chinese Medical Sciences, Nanchang, China; ^2^Jiangxi Health Industry Institute of Traditional Chinese Medicine, Nanchang, China; ^3^State Key Laboratory for Quality Ensurance and Sustainable Use of Dao-di Herbs, National Resource Center for Chinese Materia Medica, China Academy of Chinese Medical Sciences, Beijing, China; ^4^Institute of Traditional Chinese Medicine, China Academy of Chinese Medical Sciences, Beijing, China

**Keywords:** *Puerariae thomsonii*, endophytic bacteria, diversity, plant secondary metabolites, correlation analysis

## Abstract

**Introduction:**

*Puerariae thomsonii* Benth is an important medicinal and edible plant, with its dried roots being widely used in traditional Chinese medicine. The secondary metabolites of *P. thomsonii* mainly contain flavonoid compounds that have beneficial effects on human health. Current researches on the secondary metabolites of *P. thomsonii* have primarily focused on the effects of external environmental factors, while studies investigating the impact of internal microorganisms on its secondary metabolites remain limited.

**Methods:**

In this study, *P. thomsonii* roots were collected from five different regions in Jiangxi province to investigate the diversity of endophytic bacteria and their correlation with five isoflavones (puerarin, daidzin, genistin, daidzein, and genistein). The differences between endophytes and the content of five isoflavones were analyzed using high-throughput sequencing and UPLC methods. In addition, differences in endophytic bacteria across the samples from different productions were analyzed using LEfSe analysis. The functional capabilities of these bacteria were analyzed through PICRUSt2 to explore potential microbial functional traits.

**Results:**

The findings indicated that the alpha diversity of endophytic bacteria in *P. thomsonii* differed among production areas and the unique bacterial genera could be found in different areas. Four secondary metabolites in *P. thomsonii* were found to have a positive correlation with the diversity, evenness, and richness of endophytic bacterial communities using Spearman’s correlation analysis. Genera such as unclassified_f_ *Xanthomonadaceae*, *Bosea*, and *Methylobacterium–Methylorubrum* were significant positively correlated with one or more of these secondary metabolites.

**Discussion:**

This research enriches the endophytic bacterial resources of P. thomsonii, provides a preliminary analysis of the correlation between plants and microorganisms, and offers a scientific basis for the future exploration and application of endophytic resources in *P. thomsonii*.

## 1 Introduction

Endophytes are a group of microorganisms that colonize plant tissue without causing any plant diseases (Cui et al., 2024; Dang et al., 2020). Endophytes and host plants form a dynamic and balanced symbiotic relationship in the process of long-term co-evolution, which plays a crucial role in enhancing the growth, stress tolerance, and accumulation of secondary metabolites of plants. These beneficial microbes are called functional endophytes. At present, numerous studies focus on the function of endophytes and verified the beneficial effects of the functional strains on plants (Afzal et al., 2019; Kushwaha et al., 2020; Wani et al., 2015; Wu et al., 2021). Liu et al. (2022) reported that inoculating *Peucedanum praeruptorum* Dunn with a low concentration of the endophytic fungal *Didymella segeticola* can reduce the rapid decrease in coumarin content after its bolting. Abdelshafy Mohamad et al. (2020) reported that the salt stress of tomato plant and the incidence of tomato root rot can be reduced by inoculating *Bacillus* endophytes. Liu et al. (2023) found that the endophytic microbe isolated from *Taxus yunnanensis* can enhance the taxane accumulation in host stem cells.

The accumulation of secondary metabolites of medicinal plants is related to many external ecological environments, such as temperature, rainfall, and light (Li et al., 2020; Wu et al., 2024). In addition, many studies have mentioned that the quality of the medicinal plants varies with the planting origin (Dong et al., 2011; Sheng et al., 2021). Recent studies have shown that endophytic microorganisms are particularly important for the accumulation of secondary metabolites in the host plants (Kong and Glick, 2017; Lv et al., 2024). Extensive studies have employed integrated analyses of microbiome and metabolome datasets to elucidate correlations between secondary metabolites and microbial communities, thereby identifying microbial taxa with potential core applications. Correlation analyses of microbiome and metabolite content in *Alkanna tinctoria* roots revealed positive correlation between specific bacterial genera and alkannin content (Csorba et al., 2022). Yu et al. (2023) identified dominant microorganisms positively correlated with key compounds through monitoring metabolite and microbial community dynamics during Huangjiu fermentation. Furthermore, research has demonstrated that plant root exudates can modulate microbial community structures, subsequently influencing plant growth, stress resistance, and nutrient acquisition (Baker et al., 2024). Endophytic microbes are a key component of the microecosystem within medicinal plants, and they are also a rich resource to be developed. Understanding the distribution and composition of plant endophytic microbes along with their relationship with secondary metabolites of host plants can help analyze the reasons for the formation of medicinal plant quality from another perspective.

*Puerariae thomsonii* Benth, a leguminous plant widely cultivated in China, is renowned for its dried root as a traditional Chinese medicine. The ‘Chinese Pharmacopoeia’ describes that it can be used to treat influenza, body stiffness, and other illnesses (Chinese Pharmacopoeia Commission, 2020). Functioning as a medicinal and edible homologous plant resource, *P. thomsonii* is rich in starch, dietary fiber, flavonoids (primarily isoflavones), proteins, and essential mineral elements. It is consumed fresh as vegetable and processed into products such as powder extracts, functional teas, starch-based noodles, and nutraceutical pastries, with annual consumption exceeding tens of thousands of tons. Isoflavones, such as puerarin, daidzein, genistein, ononin, and their aglycones, have been reported with a lot of pharmacological benefits including antioxidant, hypoglycemic, anti-inflammatory, and anti-cancer effects (Kim, 2021; Liu et al., 2016; Wong et al., 2015). Compared with wild medicinal *Puerariae lobata*, the content of iconic isoflavones in *P. thomsonii* is lower. According to the Chinese pharmacopoeia, the minimum required puerarin content for *P. lobata* is 2.4%, whereas that for cultivated *P. thomsonii* is only 0.3% (Chinese Pharmacopoeia Commission, 2020). Narrowing this gap would enhance the medicinal value of *P. thomsonii*. Studies have shown that there are differences in the content of active components in *P. thomsonii* samples from different producing areas (Qiuyan et al., 2020; Tang et al., 2020). Endophyte communities dynamically adjust to environmental shifts, and their maintenance could be crucial for plant ecological adaptation. Endophyte-driven environmental adaptation likely shapes plant quality through secondary metabolism, yet there has been no research on the geographical association of “flora and active components” of *Pueraria* species. Under the background of the “Healthy China 2030” plan to promote the development of medicinal and edible homologous plant resource, the *P. thomsonii* industry is facing land scarcity and quality decline due to conventional fertilizer-dependent farming practices. Elucidating the interplay between endophytic microbiota and the biosynthesis of isoflavones in *P. thomsonii* holds significant implications for agricultural innovation. Furthermore, the discovery of core microorganisms with metabolic regulation function will play a key role in promoting the quality improvement of *P. thomsonii* and the development of the large-scale health industry.

*P. thomsonii* is widely planted in Jiangxi province, and in 2020, it was listed as one of the “ten flavors of Gan food” by Jiangxi province government. In this study, *P. thomsonii* samples were collected from five different production areas in Jiangxi Province. The endophytic bacterial communities were analyzed by high-throughput sequencing of the 16S rRNA gene V5-V7 region, while the contents of five isoflavones were quantified using UPLC analysis. The purposes were to evaluate the endophyte community structures that vary in different production areas and analyze the association between endophytic bacteria and the five metabolite contents in *P. thomsonii*. These findings show the ecological functions of endophytic bacteria in *P. thomsonii* and lay the groundwork for improving the quality and further development of endophytic resources in *P. thomsonii*.

## 2 Materials and methods

### 2.1 Sample collection

Between August and September 2023, healthy and fresh 1-year-old cultivated *P. thomsonii* root samples were collected from five different origins in Jiangxi province, China (Table 1). NC-region samples had four replicates while six for other regions. After being gathered in sterile plastic bags, each sample was labeled, kept in a refrigerator at 4°C, and processed within 24 h. After being repeatedly cleaned with flowing tap water until there were no more impurities, the samples were rinsed three times with sterile water. Then, 0.5 g root of each plant was took as one sample. Next, the following washing procedures were used to surface-sterilize each root sample: 75% ethanol for 1 min, sterile water for 3 times, 2.5% sodium hypochlorite solution for 5 min, followed by five final rinses using sterile water. To ensure that surface disinfection was complete, the last rinse solution was inoculated onto NA medium (incubated at 37°C for 2 d) and PDA medium (incubated at 28°C for 7 d). All samples were subsequently stored at −80°C.

**Table 1:** The information of production areas of *P. thomsonii*.

Location	Altitude (m)	Longitude	Latitude	Code
Linchuan County, Fuzhou City	70	116°30′2″	27°50′16″	LC
Nancheng Country, Fuzhou City	65	116°39′29″	27°36′26″	NC
Xingguo County, Ganzhou City	159	115°18′20″	26°9′8″	XG
Gaoan County, Yichun City	56	115°16′35″	28°27′53″	GA
Yongxiu County, Jiujiang City	22	115°46′53″	29°3′52″	YX

### 2.2 DNA extraction, amplification, and high-throughput sequencing

Genomic DNA of the root sample in *P. thomsonii* was extracted using the FastPure feces DNA Isolation Kit (Shanghai Majorbio Bio-pharm Technology Co., Ltd). The extracted DNA was tested for quality and concentration with 1.0% agarose gel electrophoresis and a NanoDrop2000 spectrophotometer (Thermo Scientific, United States) and then stored at −80°C. The V5-V7 region of the 16S rRNA gene was amplified with primer pairs 799F (5’-AACMGGATTAGATACCCKG-3′)/1392R (5’-ACGGGCGGTGTGTRC-3′) and 799F (5′-AACMGGATTAGATACCCKG -3′)/1193R (5′-ACGTCATCCCCACCTTCC -3′) in two steps by T100 Thermal Cycler PCR thermocycler (BIO-RAD, USA). The PCR reaction mixture included 10 μL 2 × Pro Taq (2 × ProTaq HS PCR Master Mix ver2, China), 0.8 μL each primer (5 μM), 4 μL of template DNA, and ddH2O to a final volume of 20 μL. Three technical replicates were performed for each reaction. The PCR amplification was performed according to the following procedure: The first step was initial denaturation at 95°C for 3 min, followed by 27 cycles of denaturing at 95°C for 30 s, annealing at 55°C for 30 s, and extension at 72°C for 45 s, and single extension at 72°C for 10 min. The second step was the same as those of the first step except 13 amplification cycles were applied. Three technical repeats of one sample were mixed as a single PCR product. The products were separated by 2% agarose gel, purified using the PCR Clean-Up Kit (YuHua, Shanghai, China) according to the manufacturer’s instructions and quantified using QuantiFluor™ -ST (Promega Biotech, Beijing, China). Purified amplicons were combined in equimolar concentrations and paired-end sequenced on an Illumina PE300/PE250 platform (Illumina, San Diego, USA) by Majorbio Bio-Pharm Technology Co. Ltd. (Shanghai, China).

### 2.3 Metabolites of PRT quantitative analysis

The standard source of puerarin, daidzin, daidzein, genistein, and genistin was Chengdu Must Biotechnology Co., Ltd. (Chengdu, Sichuan, China). Analytical-grade methanol and formic acid were offered by Xilong Scientific Company (Guangdong, China). Chromatography-grade acetonitrile was acquired from Thermo Fisher Scientific (Pittsburgh, PA, USA). Each treatment specimen’s dried root was ground into powder and sieved through a 350 μm mesh. A 0.5 g sample of the powder was precisely weighted and then mixed with 20 mL 50% methanol. The mixture was subjected to ultrasonic treatment (30–40°C, 250 W, 50 kHz) for 40 min. After cooling and restoring the sample weight, it was filtered with a 0.22 μm Millipore filter. According to sample concentration, 2–3 μL of the obtained solution was injected into the UPLC for analysis while 1 μL of the standard solution was injected into UPLC. The samples were analyzed by UPLC using CORTECS ® UPLC® C18 column (2.1 × 150 mm, 1.6 μm, Waters, USA). Chromatographic conditions were as follows: mobile phase acetonitrile (A) and 0.1% formic acid (B), elution gradient 0–3 min, 10%A; 3–15 min, 10–25%A; 15–20 min, 25–80%A; 20–20.5 min, 80–95%A; 20.5–22 min, 95%A, detection wavelength 254 nm, flow rate 2.5 mL/min, and column temperature 35°C. The following standard solutions were prepared from methanol: puerarin at 5.265 mg/mL, daidzin at 1.83 mg/mL, daidzein at 0.916 mg/mL, genistein at 0.866 mg/mL, and genistin at 0.820 mg/mL. A standard curve was then generated after each standard solution was series-diluted with methanol to the proper concentration and then tested under the previously mentioned chromatographic conditions. Sample chromatographic peaks were qualitatively determined by the standards’ retention time. The sample’s quality was assessed by standard curves.

### 2.4 Data analysis

The sequence data were processed using QIIME2. After quality filtering and chimera removal, amplicon sequence variants (ASVs) were generated using the DADA2 pipeline. Rarefaction curves were constructed to assess sequencing depth adequacy, and alpha diversity indices were calculated using Mothur v1.30.1, and the sequences were clustered at 97% similarity threshold, which is the conventional species-level cutoff for bacterial communities (Schloss et al., 2009). A Kruskal–Wallis test was employed to analyze differences in the abundance of microbial genera. Linear discriminant analysis (LDA) effect size (LEfSe) analysis (Segata et al., 2011) was performed to identify the bacterial taxa (from phylum to genera) that were significantly abundant among the different groups (LDA score > 4). Correlation analysis between host metabolites and the endophytic bacterial diversity and abundance was performed using the Spearman method. Function prediction was annotated by PICRUSt2 for the 16S rRNA ASVs. One-way analysis of variance (ANOVA) was performed using IBM SPSS Statistics 26.0. The figures in the manuscript were generated using Adobe Illustrator 2021.

## 3 Results

### 3.1 Diversity of endophytic bacteria in *Puerariae thomsonii* in different areas

The absence of colonies in the NA and PDA media culturing the last rinse of the *P. thomsonii* samples after a certain time suggested that surface sterilization was successful, and therefore, the samples were not cross-contaminated by microbes on the root surface.

**Figure 1:**
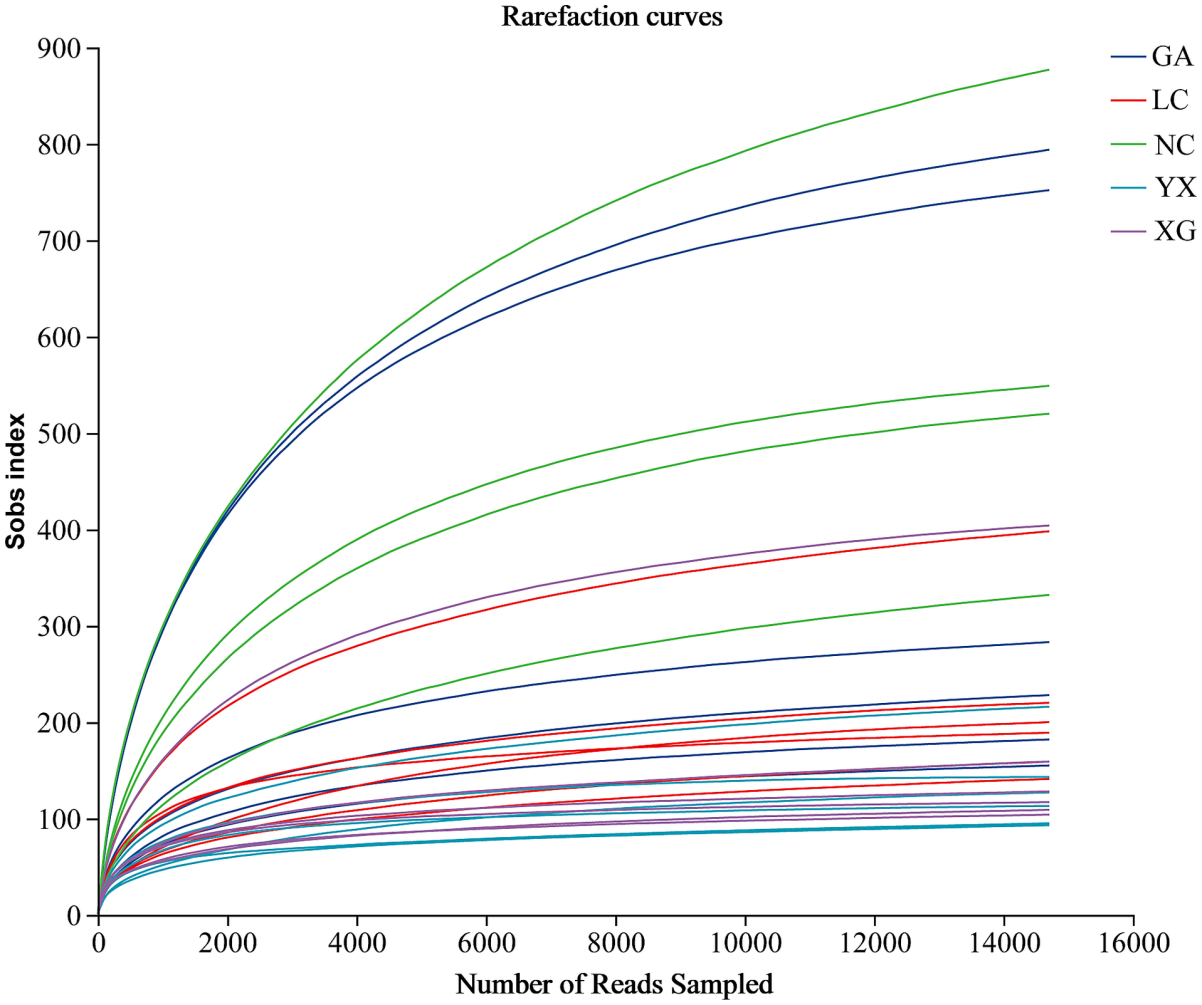
Rarefaction curves of endophytic bacteria for each sample. GA, Sample from Gaoan County, Yichun City; LC, Sample from Linchuan County, Fuzhou City; NC, Sample from Nancheng County, Fuzhou City; XG, Sample from Xingguo County, Ganzhou City; YX, Sample from Yongxiu County, Jiujiang City.

After quality control, a total of 1,516,597 high-quality sequences were generated. All of the samples’ library coverages were higher than 0.992, indicating that the sequencing data may accurately depict the endophytic bacterial community structure for each sample. The rarefaction curve can be used to assess the adequacy of sequencing data and reflect species diversity and sample richness. The rarefaction curve neared the saturation plateau with an increase in sequencing effort, indicating the sequencing depths were adequate (Figure 1).

**Figure 2:**
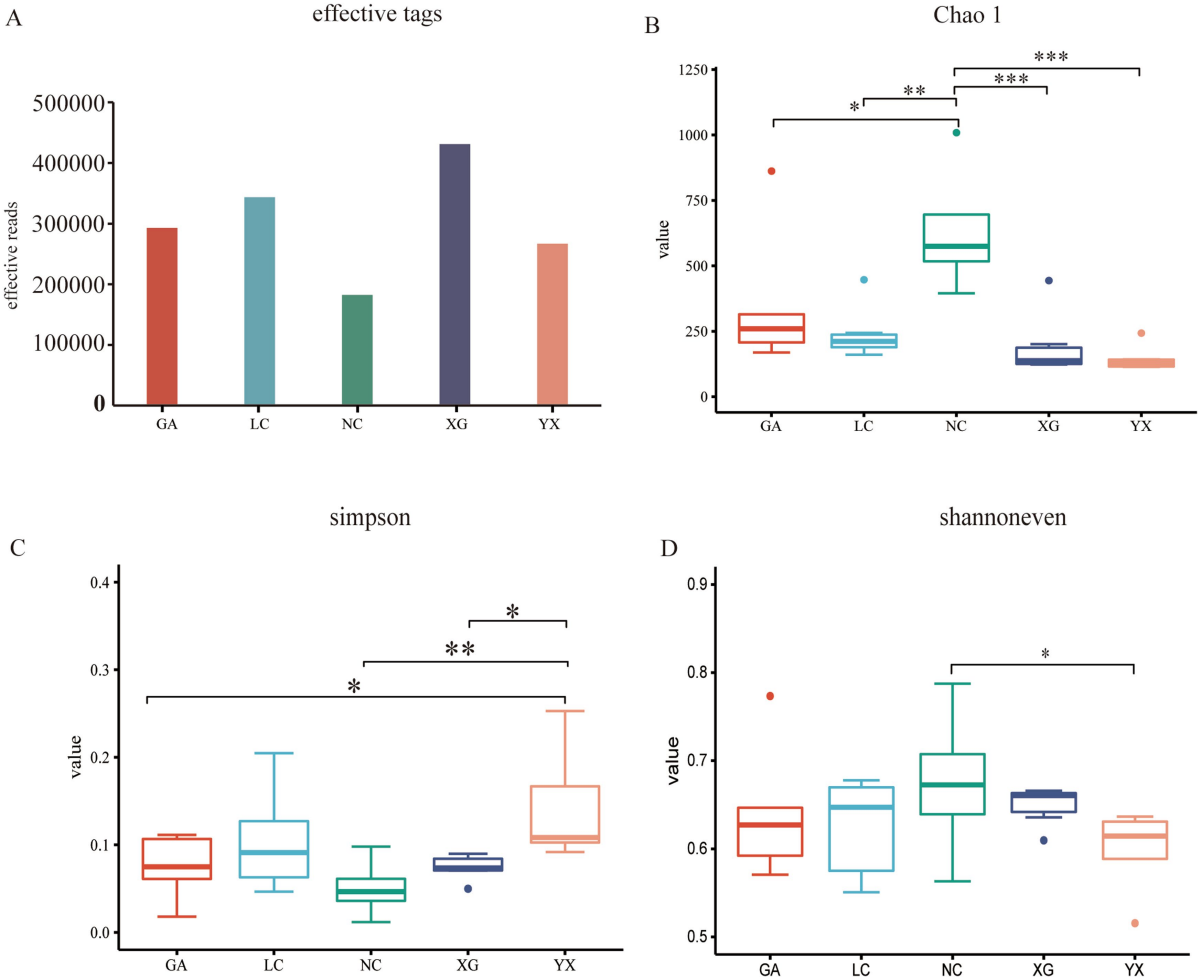
Alpha diversity index of bacterial community in *P. thomsonii* from different areas. The effective tags **(A)**, Chao 1 index **(B)**, Simpson index **(C)** and shannoneven index **(D)** of bacterial community in *P. thomsonii* from different areas. GA. Sample from Gaoan County, Yichun City; LC. Sample from Linchuan County, Fuzhou City; NC. Sample from Nancheng County, Fuzhou City; XG. Sample from Xingguo County, Ganzhou City; YX. Sample from Yongxiu County, Jiujiang City. (one-way ANOVA, *p < 0.05 and **p < 0.01, ***p < 0.001).

Alpha diversity was analyzed at the ASV level with a 97% similarity threshold. Community diversity was evaluated using multiple indices, including sample sequences (based on rarefied sequence counts), Simpson’s diversity index (measuring species dominance and community diversity), Shannon’s evenness index (quantifying species distribution uniformity in ecological communities), and the Chao1 richness estimator (assessing species richness with emphasis on rare taxa), which presented differences among all samples of *P. thomsonii*. XG had the highest number of detected sample sequences, with 430,917 sequences, while NC has the fewest sequences, with 182,382 sequences. Chao indicated a bacterial community richness trend of NC > GA > LC > XG > YX, respectively. Shannoneven index showed the sample community trends of NC > XG > GA > LC > YX, respectively. In contrast, the Simpson index showed the following trends: YX > LC > XG > GA > NC, respectively (Figure 2). This finding suggests that there were variations in the endophytic bacterial diversity, evenness, and richness among the different areas of *P. thomsonii*.

**Figure 3:**
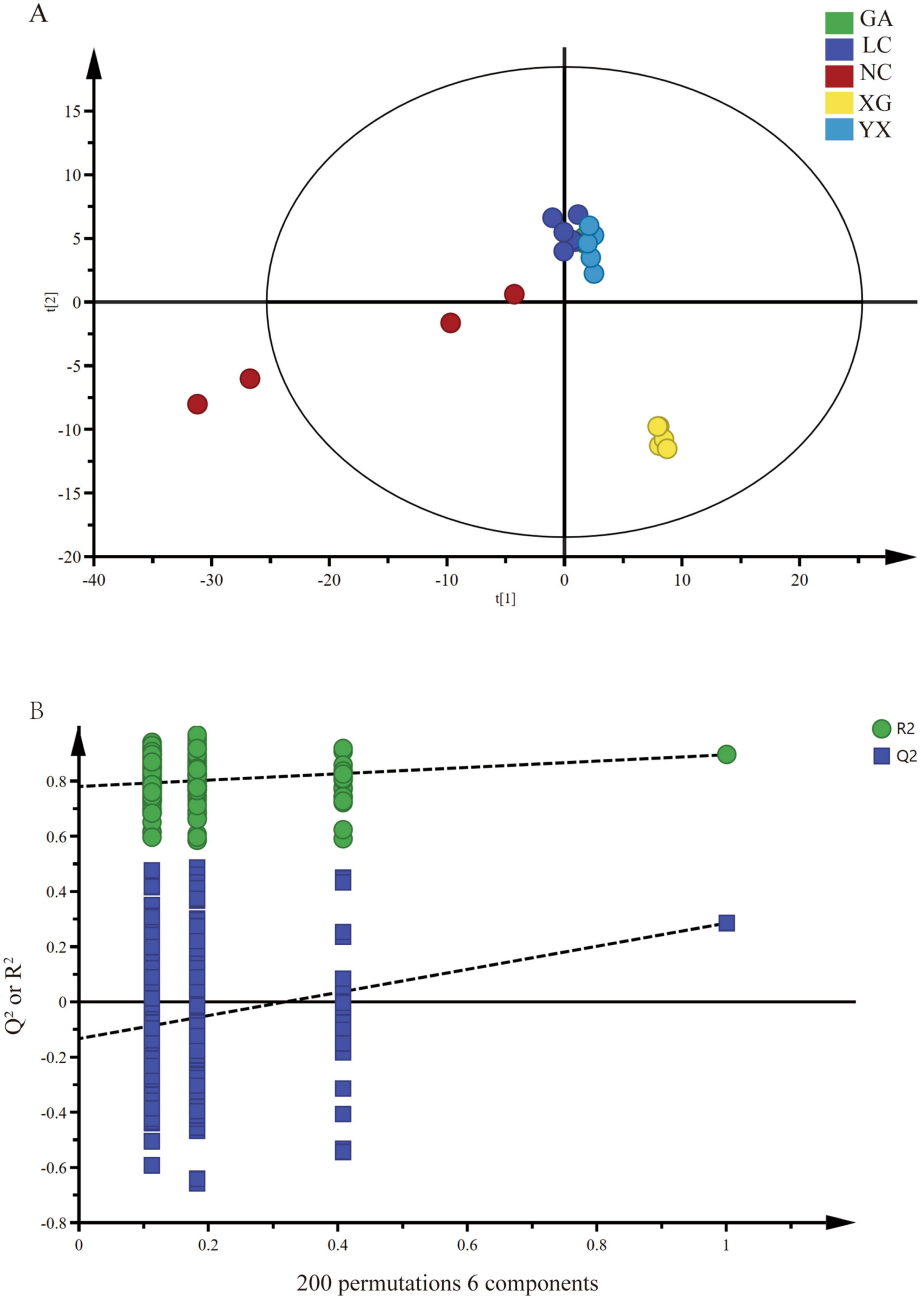
PLS-DA analysis **(A)** and permutation test **(B)** of endophytic bacteria in samples of *P. thomsonii* from different origins. GA, Sample from Gaoan County, Yichun City; LC, Sample from Linchuan County, Fuzhou City; NC, Sample from Nancheng County, Fuzhou City; XG, Sample from Xingguo County, Ganzhou City; YX, Sample from Yongxiu County, Jiujiang City.

Based on the ASV level, partial least squares analysis (PLS-DA) was performed to distinguish the endophytic bacteria of *P. thomsonii* in different origins (Figure 3). The prediction index Q^2^ = 0.511, the explanatory ability parameter of the independent variable Ry^2^ = 0.924, and the explanatory ability parameter of the dependent variable Rx^2^ = 0.582, and R^2^ and Q^2^ exceeded 0.5, indicating that the model fitting results were acceptable. Then, 200 displacement tests were carried out on the model, and the intersection point between the Q^2^ regression line and the longitudinal axis was less than 0, indicating that the model did not have overfitting, and the model verification was effective. The analysis results showed that the GA, LC, and YX were clustered into one class, and XG and NC were clustered into a separate class, respectively.

### 3.2 Community composition

**Figure 4:**
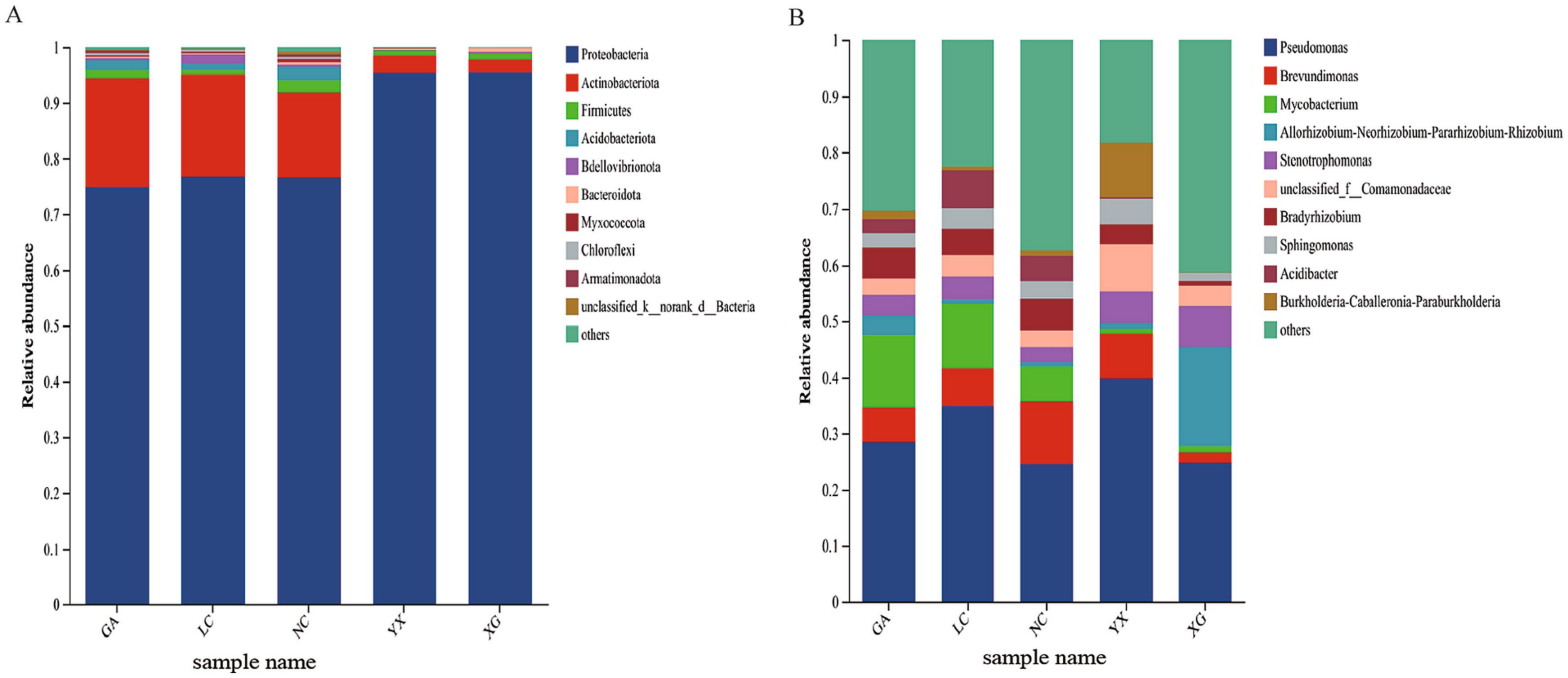
Endophytic bacteria relative abundance at the phylum level **(A)** and the genus level **(B)** in *P. thomsonii* from different production areas. “Other” represents the communities whose relative abundance outside top 10. GA, Sample from Gaoan County, Yichun City; LC, Sample from Linchuan County, Fuzhou City; NC, Sample from Nancheng County, Fuzhou City; XG, Sample from Xingguo County, Ganzhou City; YX, Sample from Yongxiu County, Jiujiang City.

The endophytic bacterial community composition of *P. thomsonii* in five plots was assigned to 27 phyla, 65 classes, 174 orders, 313 families, 634 genera, 903 species, and 4,383 ASV. Across all of the samples, Proteobacteria was the most predominant bacterial phylum exhibiting relative abundances between 74.91 and 95.84%. Furthermore, Actinobacteria constituted a significant portion of the relative abundance in the samples from GA (19.59%), LC (18.40%), and NC (15.30%) (Figure 4A). For genus level, *Pseudomonas* was the most prevalent genus in GA, LC, NC, XG, and YX samples, with relative abundances between 24.60 and 39.82%. In addition, *Allorhizobium–Neorhizobium–Pararhizobium–Rhizobium* had high abundance in XG at 17.61%, while *Mycobacterium* and *Brevundimonas* had relatively high abundance in GA, LC, and NC, accounting for 6.36 to 12.91% and 6.17 to 11.03%, respectively (Figure 4B).

**Figure 5:**
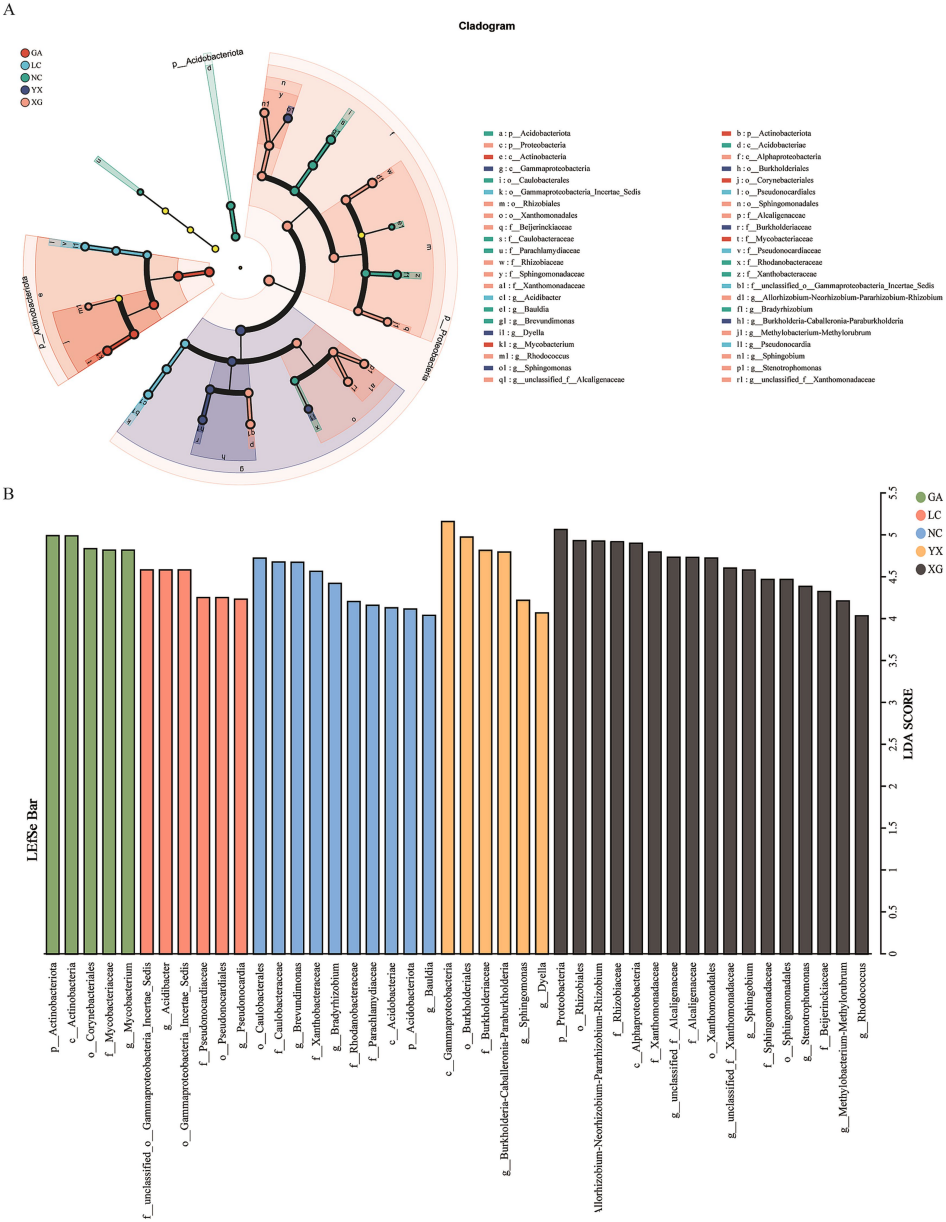
Linear discriminant effect size (LEfSe) analysis of bacterial communities at different areas. LDA score >4. In cladograms **(A)**, the circle radiating from inside to outside represents the taxonomic level from the phylum to the genus. Each small circle at a different taxonomic level represents a taxonomic at that level, and the diameter of the small circle is proportionate to the relative abundance of species. Light yellow small circle represents microbial groups with no significant differences. The LDA value distribution histogram **(B)** shows the species with significant difference in different groups. GA, Sample from Gaoan County, Yichun City; LC, Sample from Linchuan County, Fuzhou City; NC, Sample from Nancheng County, Fuzhou City; XG, Sample from Xingguo County, Ganzhou City; YX, Sample from Yongxiu County, Jiujiang City.

The endophytic bacterial biomarkers in *P. thomsonii* from different areas were identified by LEfSe, which revealed that the endophytic bacteria in *P. thomsonii* demonstrated significant differences in their cladogram structures. A total of 44 biomarkers were identified in *P. thomsonii* from different areas (LDA score > 4.0). The 44 biomarkers were distributed among the samples as follows: GA (5 taxa), LC (6 taxa), NC (10 taxa), YX (6 taxa), and XG (17 taxa). On the genus level, the GA samples contained more *Mycobacterium*, LC samples contained more *Acidibacter* and *Pseudonocardia*, NC samples contained more *Brevundimonas*, *Bradyrhizobium*, and *Bauldia*, YX samples contained more *Burkholderia–Caballeronia–Paraburkholderia*, *Sphingomonas*, and *Dyella*, and XG samples contained more *Allorhizobium–Neorhizobium–Pararhizobium–Rhizobium*, *Stenotrophomonas*, *Sphingobium*, *Methylobacterium–Methylorubrum*, *Rhodococcus*, unclassified_f_*Alcaligenaceae*, and unclassified_f_*Xanthomonadaceae* organisms than any other sample (Figure 5).

### 3.3 Correlation analysis between endophytic bacteria and the five isoflavone contents in *Puerariae thomsonii*

**Figure 6:**
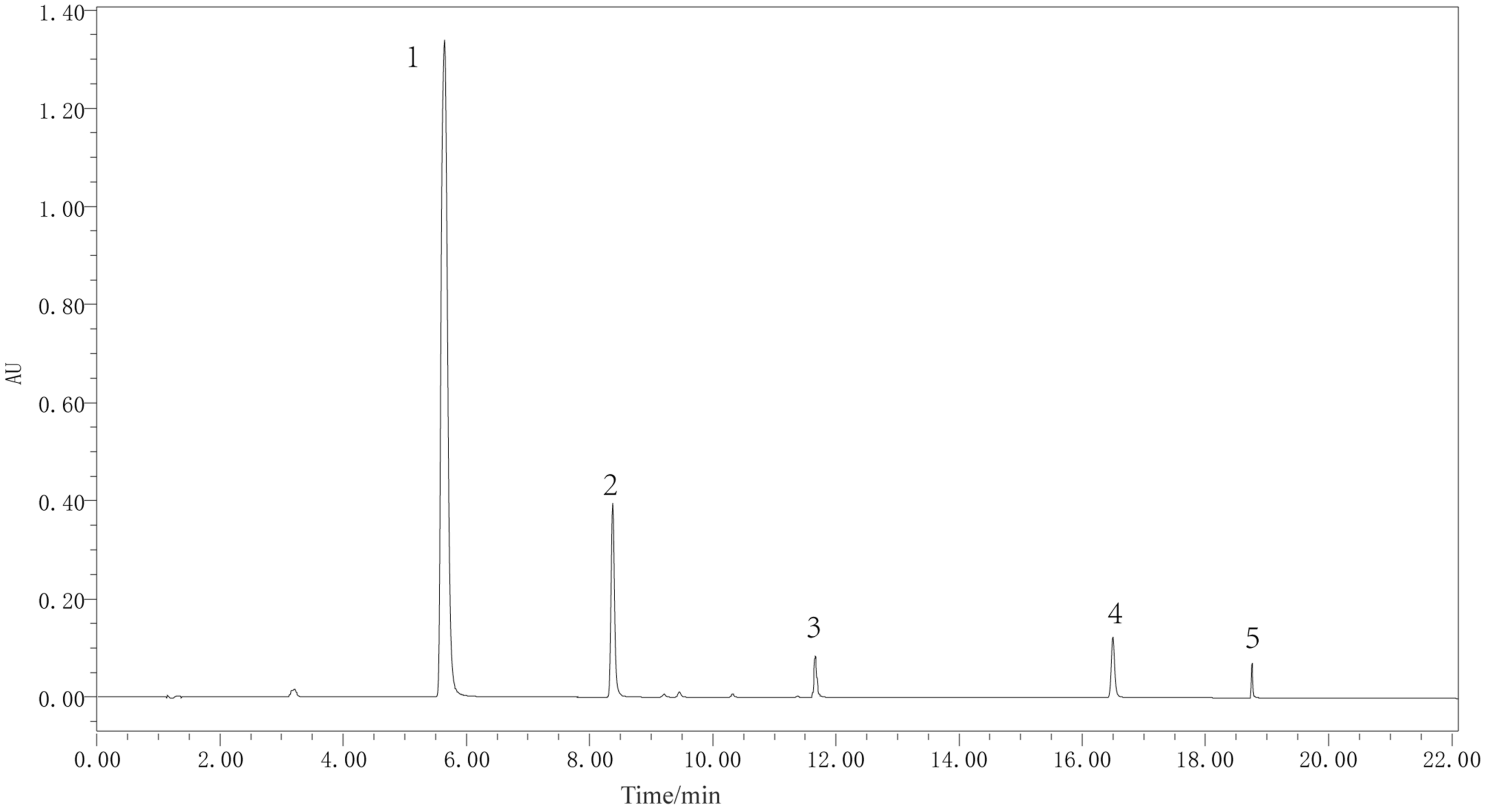
UHLC of metabolite standards of *P. thomsonii*. 1–5: puerarin, daidzin, genistin, daidzein, and genistein.

**Figure 7:**
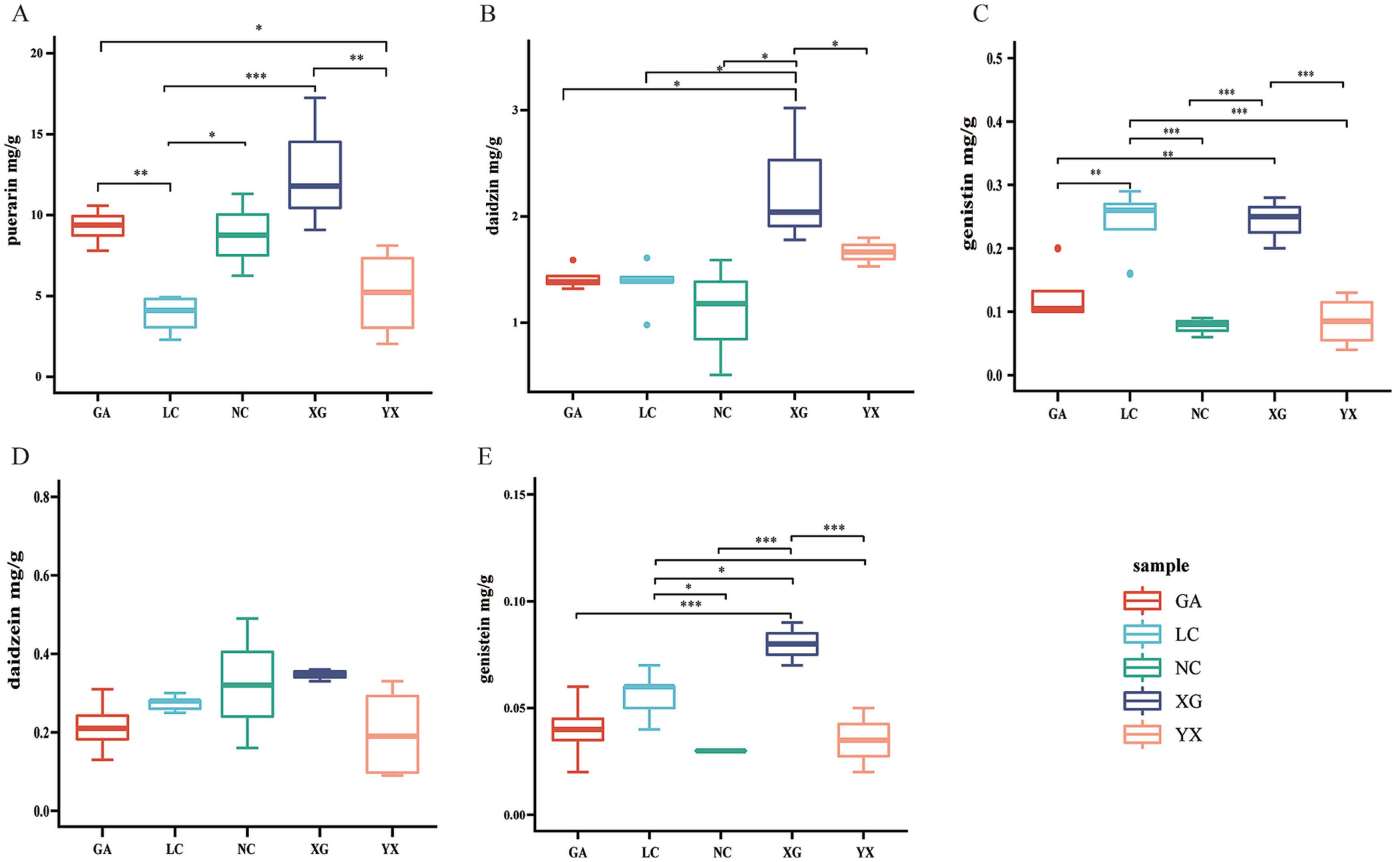
The metabolite content including puerarin **(A)**, daidzin **(B)**, genistin **(C)**, daidzein **(D)**, genistein **(E)** of P. thomsonii in different production areas. Data were shown as mean ± SD. n ≥ 3. GA. Sample from Gaoan County, Yichun City; LC. Sample from Linchuan County, Fuzhou City; NC. Sample from Nancheng County, Fuzhou City; XG. Sample from Xingguo County, Ganzhou City; YX. Sample from Yongxiu County, Jiujiang City. (One-way ANOVA, *p < 0.05 and **p < 0.01, ***p < 0.001).

**Figure 8:**
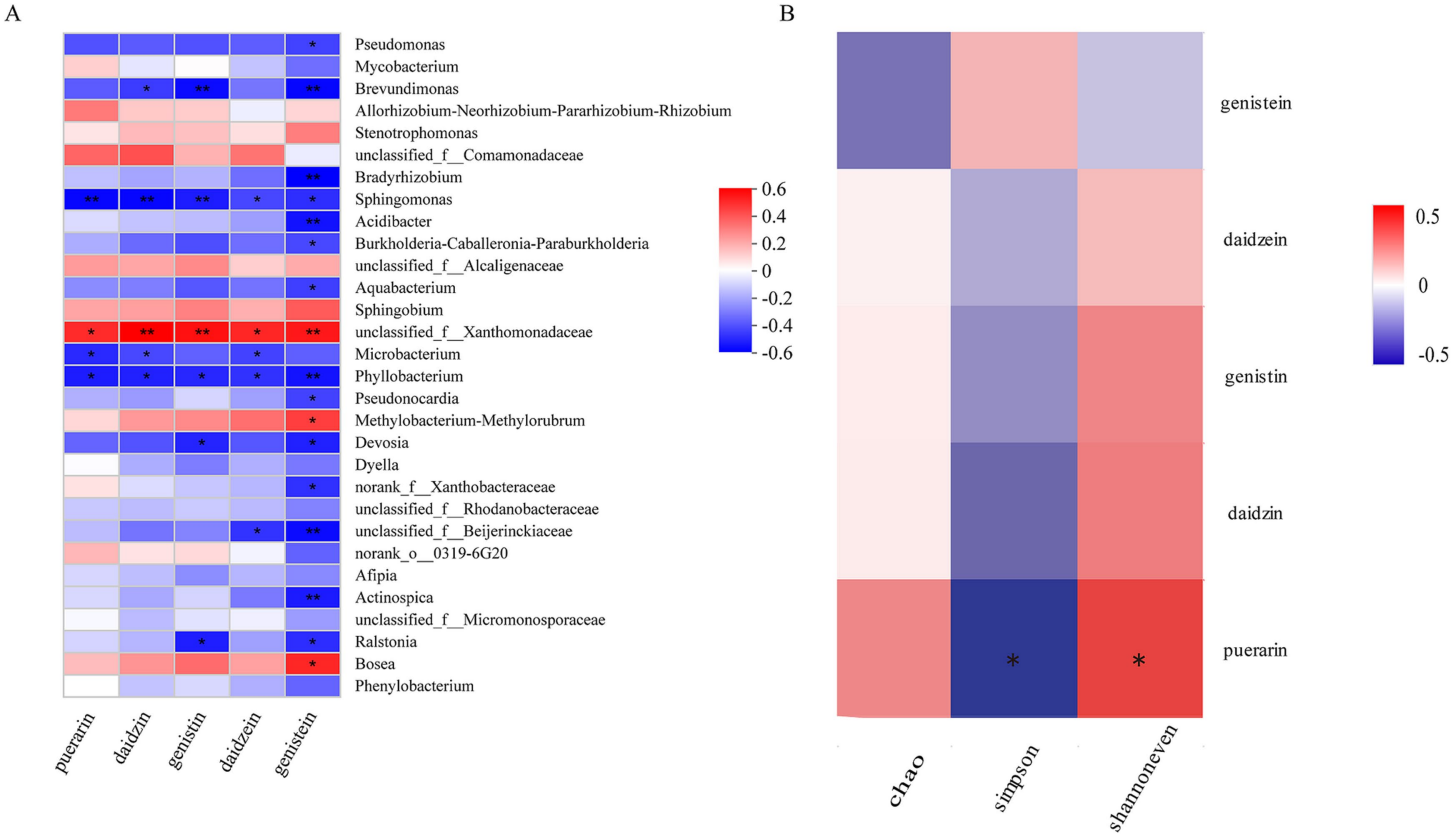
Community diversity of endophytic bacteria in *P. thomsonii* and correlation analysis of representative genera and bioactive compounds. **(A)** The heat map intuitively reflects the correlation between puerarin, daidzin, genistin, daidzein, genistein, and representative microbial genus in *P. thomsonii* (the top 30 abundances of endophytic bacteria). **(B)** Correlation analysis between puerarin, daidzin, genistin, daidzein, genistein, and the diversity of endophytes (Chao index, Simpson index, and Shannoneven index). Red indicates promotion, blue indicates inhibition, white indicates no inhibition or promotion, and the depth of color indicates the strength of inhibition or promotion. *, *p* < 0.05 and **, *p* < 0.01.

The five metabolites in *P. thomsonii* can be effectively detected under the UPLC condition (Figure 6). The content of metabolites in *P. thomsonii* differed according to their production areas (Figure 7). The Spearman heat map and co-occurrence network analysis showed the relationship between the endophytic bacterial community with the top 30 highest relative abundances and five isoflavone contents (Figure 8A, Supplementary Figure 1). The unclassified_f__*Xanthomonadaceae*, *Methylobacterium–Methylorubrum*, and *Bosea* exhibited significant positive correlations with one or more isoflavone contents in *P. thomsonii*. In addition, redundancy analysis (RDA) was also performed, in which axes 1 and axes 2 explained 21.29% of the variance of microbiome and metabolite correlation data. The results showed the accumulation of five isoflavones measured in this research showed a positive correlation with the genera of *Stenotrophomonas*, *Methylobacterium–Methylorubrum*, *Sphingobium*, unclassified_f__*Xanthomonadaceae*, unclassified_f__Alcaligenaceae, and Allorhizobium–Neorhizobium–Pararhizobium–Rhizobium (Supplementary Figure 2). Figure 8B shows the correlative patterns between the five isoflavone contents and alpha diversity metrics (Simpson index, Shannoneven, and Chao1 index). Statistical analyses revealed that both Chao1 and Shannoneven index showed inverse correlations with the genistein content, while exhibiting concordant positive trends with other four isoflavones. Among these, Shannoneven index demonstrated a moderate but statistically significant positive correlation with puerarin content (*r* = 0.378, *p* < 0.05). In contrast, the correlation coefficient of Simpson index and puerarin content was −0.45, showing a significant negative correlation (*p* < 0.05). The results suggested that samples with simple community diversity of the endophytic bacterial tentatively coincided with lower puerarin content.

### 3.4 PICRUSt2 functional prediction analysis

**Figure 9:**
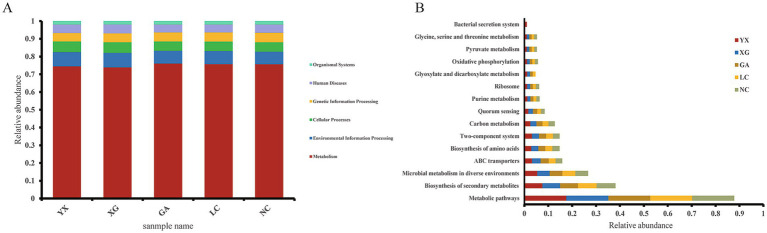
Functional annotation of endophytic bacteria by PICRUSt2. **(A)** Relative abundance of predicted KEGG Orthologs functional profiles of bacteria KEGG level 1; **(B)** relative abundance of predicted KEGG Orthologs functional profiles of bacteria KEGG level 3 (Based on the KEGG homologs whose relative abundance greater than 1%).

PICRUSt2 was utilized to forecast the functional potential of the endophytic bacterial community present in *P. thomsonii* samples from different areas based on the KEGG database (Figure 9). PICRUSt2-based predictions of potential functional traits mapped six hypothetical primary metabolic categories at the KEGG pathway level 1: metabolism, environmental information processing, cellular processes, genetic information processing, human disease, and organismal system (Figure 9A). Three hundred and eighty-nine KEGG homologs were identified. Among them, the metabolism pathway showed the highest relative prevalence across all samples, representing between 73.94 and 76.04% of the total. Based on the relative abundance greater than 1%, the prediction software PICRUSt2 highlighted 15 putatively enriched metabolic pathways at KEGG pathway level 3 (Figure 9B). Among these, metabolic pathways had a relative abundance higher than 17%, the highest in all samples; the second was the biosynthesis of secondary metabolites, with a relative abundance more than 7%, followed by microbial metabolism in diverse environments and ABC transporters, with abundances higher than 5 and 3%, respectively. The inferred pathway enrichments provide testable hypotheses that warrant further validation through targeted metatranscriptomics or biochemical assays.

## 4 Discussion

### 4.1 Geographic distribution of *Puerariae thomsonii* isoflavones and endophytes

The content of active ingredients in medicinal plants is influenced by the cultivation site, and the content of their active components is closely related to the quality of the medicinal materials. In this study, samples from five different origins of *P. thomsonii* were collected from Jiangxi Province for the determination of the content of five isoflavones. The results showed that the active components of the samples from different origins were quite different and showed significant differences in different production areas except for daidzein. Among all the origins, the content of the five components in XG was relatively high, while in other origins, the components showed different distribution patterns (Figure 7). Zhao S. et al. (2023) found that the contents of rhein, sennoside A, and gallic acid in wild *Rheum tanguticum* were relatively higher in Qinghai province than in Sichuan and Gansu province. The metabolome analysis of *Cynomorium songaricum* collected from different production origins showed that the metabolic components of the sample from different regions showed obvious differences (Cui et al., 2019). The content of active ingredients in medicinal plant varied among different origins, which may be related to the environment or microbial difference in different origins. The endophytic bacteria in *P. thomsonii* from different production origins were sequenced by high-throughput sequencing. Alpha diversity analysis showed that the richness, diversity, and evenness of the endophytic bacterial community from the NC region were at a high level, while the YX region was low. The diversity of endophytic bacteria in *P. thomsonii* from different origins showed significant differences, consistent with the research in *Polygonatum cyrtonema* Hua (Zhang et al., 2024) and *Gastrodia elata* f. glauca (Zheng H. et al., 2023), which may be due to the differences in geographical environment. The difference in endophytic bacteria from different origin may be one of the reasons for the difference of active ingredients of *P. thomsonii*.

### 4.2 Regional variation in endophytic bacterial communities of *Puerariae thomsonii*

Endophytes are mutually beneficial and symbiotic with the host plant and play a vital role in the process of plant growth. Across all samples in this study, Proteobacteria was the predominant phyla of bacterial endophyte, exhibiting a relative abundance of more than 75% in each sample, followed by Actinobacteriota. Previous studies have reported similar results observed in many plants (Chen et al., 2024; Hou et al., 2022), as well as in the rhizosphere soil (Chen J. et al., 2021; Sun et al., 2022), because the rhizosphere microbiome is an important source of plant endophytes (Miranda-Carrazco et al., 2022). At the genus level, *Pseudomonas* presented as the predominant bacterial genus, the relative abundances ranging from 24.60 to 39.82% across all sample (Figure 4B), followed by *Allorhizobium–Neorhizobium–Pararhizobium–Rhizobium*, *Mycobacterium*, and *Brevundimonas*. The genera composition of *P. thomsonii* samples from different origin was similar, but there were some differences in abundance. By differential discriminant analysis of endophytic bacteria from different origins, 44 biomarkers of endophytic bacteria from different origin were obtained, including 16 biomarkers at the genus level (LDA score >4.0). Most are reported to enhance environmental remediation, plant growth, stress tolerance, and secondary metabolite accumulation. *Stenotrophomonas* sp. has been shown to have a good nitrogen fixation capacity and can promote the growth of barley, wheat, maize, and other crops (Zhou et al., 2025). After inoculated with *Sphingobium* bacteria, the plant height, leaf size, and chlorophyll content of *Pinellia ternata* increased obviously (Li J. et al., 2024). Li C. et al. (2024) found that pre-inoculation of *Bradyrhizobium japonicum* strain enhanced salt tolerance in soybean plants and suggested that GmADC may be a key gene to play this role. Some strains of *Sphingomonas* have the functions of repairing environmental pollution, producing plant hormones, improving plant stress, and so on (Asaf et al., 2020). These results indicated that the endophytic bacteria of *P. thomsonii* were basically beneficial microorganisms. Soil microbial communities vary across production areas, and plants selectively recruit these communities during their growth to improve fitness and stress resilience. Therefore, even for the same plant, the endophytic microbiota formed in different places of origin is probably different. Yang et al. (2023) showed that planting location significantly affected the bacterial community composition in different organs of soybean. Jiang M. et al. (2024) showed that soil type could affect the synthesis of *Angelica dahurica* var. *formosana* root exudates, thereby recruiting beneficial microorganisms and leading to differences in rhizosphere microorganisms in different producing areas.

### 4.3 Correlation analysis between endophytic bacteria and isoflavone contents across *Puerariae thomsonii* origins

Many studies reported that endophytic microbiomes significantly influence plant overall health and functionality. The endophytes can not only enhance the generation of host plant secondary metabolites (Qianliang et al., 2013; Sucheta et al., 2021) but also synthesize chemical substances that are identical or similar to the host plant (Zhao et al., 2011). To investigate such interactions, we analyzed correlation between *P. thomsonii* endophytic bacterial communities and its isoflavone content. Spearman’s correlation analysis revealed statistical correlation between specific bacterial taxa and isoflavones, revealing multiple genera positively correlated with the isoflavones measured in this research. Notably, the unclassified_f__*Xanthomonadaceae* genus showed a significant positive correlation with all five isoflavone contents measured in this study (*p* < 0.05), while *Bosea* and *Methylobacterium–Methylorubrum* displayed significant positive correlations with genistin content specifically (*p* < 0.05). These findings aligned with redundancy analysis (RDA), which showed positive correlation of five isoflavones accumulation with the *Stenotrophomonas*, *Methylobacterium–Methylorubrum*, *Sphingobium*, unclassified_f__*Xanthomonadaceae*, unclassified_f__*Alcaligenaceae,* and *Allorhizobium–Neorhizobium–Pararhizobium–Rhizobium*. The role of microorganisms in the regulation of plant secondary metabolism has been widely recognized. Specific microorganisms can increase the synthesis of plant secondary metabolites, and the beneficial effects depend on environmental factors and the microorganisms associated with the plant (Lv et al., 2024). Earlier research studies have also indicated that some strains in *Xanthomonadaceae*, *Bosea*, and *Methylobacterium* are plant growth-promoting bacteria. After inoculation with *Xanthomonadaceae* strains, the root length and root surface area of *Medicago truncatula* were significantly increased, and the growth was also promoted (Kępczyńska and Karczyński, 2019). Zheng Q. et al. (2023) found that inoculating with strain *Bosea* sp_L1B56 could significantly increase the total biomass production of *Brachypodium distachyon*. *Methylorubrum extorquens* AM1 can be used to produce violacein (Quynh Le et al., 2022), and vaccination with *Methylorubrum rhodesianum* M520 can reduce root knot nematodes in cucumber (Zhao Z. et al., 2023). No direct evidence currently links these taxa to isoflavone biosynthesis in the present system. The observed correlations may reflect ecological preferences rather than causal mediation and need to be verified by follow-up experiments, which is where we will go next. In our study, the recruitment of these microorganisms (unclassified _f__ *Xanthomonadaceae* genus, *Bosea* genus, and *Methylobacterium–Methylorubrum* genus), which exhibited significant positive correlation with the secondary metabolites, may promote the biosynthesis and accumulation of isoflavones in *P. thomsonii*. However, the potential role of these bacteria in promoting the accumulation of secondary metabolites in *P. thomsonii* remains to be explored of our subsequent research.

For endophytic bacterial diversity, we found that the content of puerarin, daidzin, genistin, and daidzein had a positive correlation with the community diversity, evenness, and richness of *P. thomsonii*. Puerarin content was significantly negatively correlated with Simpson index and positively correlated with Shannoneven index, suggesting a tentative link between community evenness, diversity, and puerarin accumulation. Among all the producing areas, the endophytic bacterial community diversity and evenness of samples from YX were the lowest, and the puerarin content was 5.15 mg/g; the endophytic bacterial community diversity and evenness of samples from NC were the highest, and the puerarin content was 8.77 mg/g. However, such patterns were not universally predictive. With the increase of microbial diversity, the microbial community functions such as nutrient mineralization or disease inhibition were also enhanced (Saleem et al., 2019). Maize intercropping with other crops can significantly increase rhizosphere microbiome diversity, thus promoting maize growth and nutrient absorption (Jiang P. et al., 2024). According to the study of Chen D. et al. (2021), the diversity of endophytic bacteria in *Rheum palmatum* from different production areas was negatively correlated with the contents of aloe-emodin, rhein, emodin, chrysophanol, and physcion. This is contrary to our results, suggesting that the effects of endophyte diversity on host secondary metabolites may vary with plants. In our study, the diversity of endophytic bacteria was correlated with the secondary metabolic components, but it was not the case that the higher the diversity of endophytic bacteria, the higher the content of secondary metabolites. It is speculated that the impact of microbial diversity on plants not only varies according to host species but also is affected by other factors such as the abundance of beneficial bacteria.

## 5 Conclusion

In this study, the endophytic microbial community structure and the contents of five isoflavones in *P. thomsonii* were investigated in different production areas, and the correlation between endophytic bacteria and active ingredients was clarified. This study revealed significant differences in both isoflavone content and endophytic bacterial composition among *P. thomsonii* samples collected from different production areas. In addition, distinct microbial communities were observed across different production regions. Correlation analysis showed that the unclassified _f__ *Xanthomonas* genus, *Bosea* genus, and *Methylobacterium–Methylorubrum* genus were significantly positively correlated with the content of one or more isoflavone components, suggesting tentative links that these taxa could sever as potential contributors to metabolite variation. However, the function of these bacterial genera in *P. thomsonii* needs to be further verified, and they are expected to serve as a raw material for the development of microbial fertilizers.

## Data Availability

The datasets for this study can be found in the Genome Sequence Archive (Genomics, Proteomics & Bioinformatics 2021) in National Genomics Data Center (Nucleic Acids Res 2022), China National Center for Bioinformation / Beijing Institute of Genomics, Chinese Academy of Sciences (GSA: CRA020510) that are publicly accessible at https://ngdc.cncb.ac.cn/gsa.

## Supplementary material

SUPPLEMENTARY FIGURE 1 Association network of endophytic bacteria and metabolites in *P. thomsonii* samples. Genera with top 30 relative abundance were selected. Pearson correlation coefficients (|*r*| >0.3; *p* < 0.05) between bacterial genera and metabolites were calculated. The size of the node in the network is related to the connective degree of the point; the color represents the phylogenetic phyla for bacteria and chemical classes for metabolites; the red and green lines represent the positive and negative correlations between the two nodes, respectively; the thicker the line, the stronger is the correlation.

SUPPLEMENTARY FIGURE 2 Redundancy analysis (RDA) of dominant microorganisms and metabolites in *P. thomsonii* samples. The red arrows represent different metabolites, and the blue arrows represent dominant microorganisms. Circles in different color represent samples from GA, LC, NC, XG, and YX region, respectively.

## References

[ref1] Abdelshafy Mohamad O. A. Ma J. B. Liu Y. H. Zhang D. Hua S. Bhute S.. (2020). Beneficial endophytic bacterial populations associated with medicinal plant *Thymus vulgaris* alleviate salt stress and confer resistance to fusarium oxysporum. Front. Plant Sci. 11:47. doi: 10.3389/fpls.2020.00047, PMID: 32117385 PMC7033553

[ref2] Afzal I. Shinwari Z. K. Sikandar S. Shahzad S. (2019). Plant beneficial endophytic bacteria: mechanisms, diversity, host range and genetic determinants. Microbiol. Res. 221, 36–49. doi: 10.1016/j.micres.2019.02.001, PMID: 30825940

[ref3] Asaf S. Numan M. Khan A. L. Al-Harrasi A. (2020). Sphingomonas: from diversity and genomics to functional role in environmental remediation and plant growth. Crit. Rev. Biotechnol. 40, 138–152. doi: 10.1080/07388551.2019.1709793, PMID: 31906737

[ref4] Baker N. R. Zhalnina K. Yuan M. Herman D. Ceja-Navarro J. A. Sasse J.. (2024). Nutrient and moisture limitations reveal keystone metabolites linking rhizosphere metabolomes and microbiomes. Proc. Natl. Acad. Sci. U. S. A. 121:e2303439121. doi: 10.1073/pnas.2303439121, PMID: 39093948 PMC11317588

[ref5] Chen D. Jia L. Hou Q. Zhao X. Sun K. (2021). Analysis of endophyte diversity of *Rheum palmatum* from different production areas in Gansu Province of China and the association with secondary metabolite. Microorganisms 9:978. doi: 10.3390/microorganisms9050978, PMID: 33946518 PMC8147242

[ref6] Chen J. Li N. Chang J. Ren K. Zhou J. Yang G. E. (2021). Taxonomic structure of rhizosphere bacterial communities and its association with the accumulation of alkaloidal metabolites in *Sophora flavescens*. Front. Microbiol. 12:781316. doi: 10.3389/fmicb.2021.781316, PMID: 34970241 PMC8712762

[ref7] Chen X. Li L. He Y. (2024). Epiphytic and endophytic bacteria on Camellia oleifera phyllosphere: exploring region and cultivar effect. BMC Ecol. Evol. 24:62. doi: 10.1186/s12862-024-02240-3, PMID: 38735962 PMC11089727

[ref8] Chinese Pharmacopoeia Commission (2020). The Chinese Pharmacopoeia. Beijing, China: The chemical industry publishing house.

[ref9] Csorba C. Rodić N. Zhao Y. Antonielli L. Brader G. Vlachou A.. (2022). Metabolite production in *Alkanna tinctoria* links plant development with the recruitment of individual members of microbiome thriving at the root-soil Interface. mSystems 7:e0045122. doi: 10.1128/msystems.00451-22, PMID: 36069453 PMC9601132

[ref10] Cui J. L. Gong Y. Vijayakumar V. Zhang G. Wang M. L. Wang J. H.. (2019). Correlation in chemical metabolome and endophytic Mycobiome in Cynomorium songaricum from Different Desert locations in China. J. Agric. Food Chem. 67, 3554–3564. doi: 10.1021/acs.jafc.9b00467, PMID: 30860831

[ref11] Cui S. Zhou L. Fang Q. Xiao H. Jin D. Liu Y. (2024). Growth period and variety together drive the succession of phyllosphere microbial communities of grapevine. Sci. Total Environ. 950:175334. doi: 10.1016/j.scitotenv.2024.175334, PMID: 39117232

[ref12] Dang H. Zhang T. Li G. Mu Y. Lv X. Wang Z.. (2020). Root-associated endophytic bacterial community composition and structure of three medicinal licorices and their changes with the growing year. BMC Microbiol. 20:291. doi: 10.1186/s12866-020-01977-3, PMID: 32957914 PMC7507641

[ref13] Dong J. Ma X. Wei Q. Peng S. Zhang S. (2011). Effects of growing location on the contents of secondary metabolites in the leaves of four selected superior clones of *Eucommia ulmoides*. Ind. Crop. Prod. 34, 1607–1614. doi: 10.1016/j.indcrop.2011.06.007

[ref14] Hou Q. Z. Chen D. W. Wang Y. P. Ehmet N. Ma J. Sun K. (2022). Analysis of endophyte diversity of two Gentiana plants species and the association with secondary metabolite. BMC Microbiol. 22:90. doi: 10.1186/s12866-022-02510-4, PMID: 35392806 PMC8988345

[ref15] Jiang P. Wang Y. Zhang Y. Fei J. Rong X. Peng J.. (2024). Intercropping enhances maize growth and nutrient uptake by driving the link between rhizosphere metabolites and microbiomes. New Phytol. 243, 1506–1521. doi: 10.1111/nph.19906, PMID: 38874414

[ref16] Jiang M. Zhang K. He L. Liu S. Liu R. Zhang Y.. (2024). The flavonoids in root regulated rhizosphere microbiome of Angelica dahurica var. formosana in genuine producing area. Ind. Crop. Prod. 219:119164. doi: 10.1016/j.indcrop.2024.119164

[ref17] Kępczyńska E. Karczyński P. (2019). *Medicago truncatula* root developmental changes by growth-promoting microbes isolated from Fabaceae, growing on organic farms, involve cell cycle changes and WOX5 gene expression. Planta 251:25. doi: 10.1007/s00425-019-03300-5, PMID: 31784832

[ref18] Kim I. S. (2021). Current perspectives on the beneficial effects of soybean Isoflavones and their metabolites for humans. Antioxidants 10:1064. doi: 10.3390/antiox10071064, PMID: 34209224 PMC8301030

[ref19] Kong Z. Glick B. R. (2017). The role of plant growth-promoting Bacteria in metal phytoremediation. Adv. Microb. Physiol. 71, 97–132. doi: 10.1016/bs.ampbs.2017.04.001, PMID: 28760324

[ref20] Kushwaha P. Kashyap P. L. Bhardwaj A. K. Kuppusamy P. Srivastava A. K. Tiwari R. K. (2020). Bacterial endophyte mediated plant tolerance to salinity: growth responses and mechanisms of action. World J. Microbiol. Biotechnol. 36:26. doi: 10.1007/s11274-020-2804-9, PMID: 31997078

[ref21] Li C. Huang Q. Sun S. Cheng C. Chen Y. Yu B. (2024). Preinoculation with *Bradyrhizobium japonicum* enhances the salt tolerance of *Glycine max* seedlings by regulating polyamine metabolism in roots. Plant Physiol. Biochem. 216:109196. doi: 10.1016/j.plaphy.2024.109196, PMID: 39405999

[ref22] Li Y. Kong D. Fu Y. Sussman M. R. Wu H. (2020). The effect of developmental and environmental factors on secondary metabolites in medicinal plants. Plant Physiol. Biochem. 148, 80–89. doi: 10.1016/j.plaphy.2020.01.006, PMID: 31951944

[ref23] Li J. Qu K. Wei L. Chen H. Cai H. Zhang J.. (2024). Artemisia argyi leaf powder improves soil properties and recruits Sphingobium bacteria to promote the growth and yield of *Pinellia ternata*. J. Environ. Manag. 371:123322. doi: 10.1016/j.jenvman.2024.123322, PMID: 39547026

[ref24] Liu Q. Li L. Chen Y. Wang S. Xue L. Meng W.. (2023). Diversity of endophytic microbes in Taxus yunnanensis and their potential for plant growth promotion and Taxane accumulation. Microorganisms 11:1645. doi: 10.3390/microorganisms11071645, PMID: 37512818 PMC10383522

[ref25] Liu C. M. Ma J. Q. Liu S. S. Feng Z. J. Wang A. M. (2016). Puerarin protects mouse liver against nickel-induced oxidative stress and inflammation associated with the TLR4/p38/CREB pathway. Chem. Biol. Interact. 243, 29–34. doi: 10.1016/j.cbi.2015.11.017, PMID: 26607348

[ref26] Liu L. Wang X. Chen S. Liu D. Song C. Yi S.. (2022). Fungal isolates influence the quality of Peucedanum praeruptorum Dunn. Front. Plant Sci. 13:1011001. doi: 10.3389/fpls.2022.1011001, PMID: 36352875 PMC9638934

[ref27] Lv J. Yang S. Zhou W. Liu Z. Tan J. Wei M. (2024). Microbial regulation of plant secondary metabolites: impact, mechanisms and prospects. Microbiol. Res. 283:127688. doi: 10.1016/j.micres.2024.127688, PMID: 38479233

[ref28] Miranda-Carrazco A. Navarro-Noya Y. E. Govaerts B. Verhulst N. Dendooven L. (2022). Nitrogen fertilizer application alters the root endophyte bacterial microbiome in maize plants, but not in the stem or rhizosphere soil. Microbiol. Spectr. 10:e0178522. doi: 10.1128/spectrum.01785-22, PMID: 36255324 PMC9769722

[ref29] Qianliang M. Chunyan S. Chengjian Z. Min J. Qiaoyan Z. Hong Z.. (2013). Elicitors from the endophytic fungus Trichoderma atroviride promote *Salvia miltiorrhiza* hairy root growth and tanshinone biosynthesis. J. Exp. Bot. 64, 5687–5694. doi: 10.1093/jxb/ert342, PMID: 24127517

[ref30] Qiuyan M. Jinzhou H. Yanlin L. Yanfang T. Yanhua W. (2020). Comparison of polysaccharides and Isoflavones in *Pueraria thomsonii* and *Pueraria lobata* from different producing areas of Guangxi. Food Res Dev 41, 43–50.

[ref31] Quynh Le H. T. Anh Mai D. H. Na J. G. Lee E. Y. (2022). Development of Methylorubrum extorquens AM1 as a promising platform strain for enhanced violacein production from co-utilization of methanol and acetate. Metab. Eng. 72, 150–160. doi: 10.1016/j.ymben.2022.03.008, PMID: 35301124

[ref32] Saleem M. Hu J. Jousset A. (2019). More than the sum of its parts: microbiome biodiversity as a driver of plant growth and soil health. Annu. Rev. Ecol. Evol. Syst. 50, 145–168. doi: 10.1146/annurev-ecolsys-110617-062605

[ref33] Schloss P. D. Westcott S. L. Ryabin T. Hall J. R. Hartmann M. Hollister E. B.. (2009). Introducing mothur: open-source, platform-independent, community-supported software for describing and comparing microbial communities. Appl. Environ. Microbiol. 75, 7537–7541. doi: 10.1128/AEM.01541-09, PMID: 19801464 PMC2786419

[ref34] Segata N. Izard J. Waldron L. Gevers D. Miropolsky L. Garrett W. S.. (2011). Metagenomic biomarker discovery and explanation. Genome Biol. 12:R60. doi: 10.1186/gb-2011-12-6-r60, PMID: 21702898 PMC3218848

[ref35] Sheng Z. Liu J. Yang B. (2021). Structure differences of water soluble polysaccharides in Astragalus membranaceus induced by origin and their bioactivity. Food Secur. 10:1755. doi: 10.3390/foods10081755, PMID: 34441532 PMC8395020

[ref36] Sucheta S. S. P. S. Rashmi T. Alok P. Karuna S. Alok K. (2021). Endophytic consortium with growth-promoting and alkaloid enhancing capabilities enhance key terpenoid indole alkaloids of *Catharanthus roseus* in the winter and summer seasons. Ind. Crop. Prod. 166:113437. doi: 10.1016/j.indcrop.2021.113437, PMID: 40275114

[ref37] Sun H. Shao C. Jin Q. Li M. Zhang Z. Liang H.. (2022). Response of microbial community structure to chromium contamination in *Panax ginseng*-growing soil. Environ. Sci. Pollut. Res. 29, 61122–61134. doi: 10.1007/s11356-022-20187-0, PMID: 35435557

[ref38] Tang Z. Wang Y. Yang J. Xiao Y. Cai Y. Wan Y.. (2020). Isolation and identification of flavonoid-producing endophytic fungi from medicinal plant Conyza blinii H.Lév that exhibit higher antioxidant and antibacterial activities. PeerJ 8:e8978. doi: 10.7717/peerj.8978, PMID: 32328352 PMC7166047

[ref39] Wani Z. A. Ashraf N. Mohiuddin T. Riyaz-Ul-Hassan S. (2015). Plant-endophyte symbiosis, an ecological perspective. Appl. Microbiol. Biotechnol. 99, 2955–2965. doi: 10.1007/s00253-015-6487-3, PMID: 25750045

[ref40] Wong K. H. Razmovski-Naumovski V. Li K. M. Li G. Q. Chan K. (2015). Comparing morphological, chemical and anti-diabetic characteristics of Puerariae Lobatae Radix and Puerariae Thomsonii Radix. J. Ethnopharmacol. 164, 53–63. doi: 10.1016/j.jep.2014.12.050, PMID: 25560667

[ref41] Wu W. Chen W. Liu S. Wu J. Zhu Y. Qin L.. (2021). Beneficial relationships between endophytic Bacteria and medicinal plants. Front. Plant Sci. 12:6146. doi: 10.3389/fpls.2021.646146, PMID: 33968103 PMC8100581

[ref42] Wu Y. Zhuo Z. Qian Q. Xu D. (2024). Chemotaxonomic variation of volatile components in Zanthoxylum Bungeanum peel and effects of climate on volatile components. BMC Plant Biol. 24:793. doi: 10.1186/s12870-024-05485-8, PMID: 39169301 PMC11340169

[ref43] Yang H. Ye W. Yu Z. Shen W. Li S. Wang X.. (2023). Host niche, genotype, and field location shape the diversity and composition of the soybean microbiome. J. Integr. Agric. 22, 2412–2425. doi: 10.1016/j.jia.2023.01.006

[ref44] Yu H. Li Q. Guo W. Chen C. Ai L. Tian H. (2023). Dynamic analysis of volatile metabolites and microbial community and their correlations during the fermentation process of traditional Huangjiu (Chinese rice wine) produced around winter solstice. Food Chem. X 18:100620. doi: 10.1016/j.fochx.2023.100620, PMID: 36993869 PMC10041457

[ref45] Zhang Q. Cai Y. Zhang L. Lu M. Yang L. Wang D.. (2024). The accumulation of active ingredients of Polygonatum cyrtonema Hua is associated with soil characteristics and bacterial community. Front. Microbiol. 15:7204. doi: 10.3389/fmicb.2024.1347204, PMID: 38559348 PMC10978593

[ref46] Zhao J. Shan T. Mou Y. Zhou L. (2011). Plant-derived bioactive compounds produced by endophytic fungi. Mini Rev. Med. Chem. 11, 159–168. doi: 10.2174/138955711794519492, PMID: 21222580

[ref47] Zhao Z. Wang L. Khan R. A. A. Song X. Najeeb S. Zhao J.. (2023). Methylorubrum rhodesianum M520 as a biocontrol agent against Meloidogyne incognita (Tylenchida: Heteroderidae) J2s infecting cucumber roots. J. Appl. Microbiol. 134:lxad001. doi: 10.1093/jambio/lxad001, PMID: 36611228

[ref48] Zhao S. Xiong F. Wang L. Wang B. Chen K. Chen C.. (2023). Study on the quality characteristics and geographical origin authentication of wild Rheum tanguticum in three authentic regions. J. Food Compos. Anal. 123:105463. doi: 10.1016/j.jfca.2023.105463

[ref49] Zheng Q. Hu Y. Kosina S. M. Van Goethem M. W. Tringe S. G. Bowen B. P.. (2023). Conservation of beneficial microbes between the rhizosphere and the cyanosphere. New Phytol. 240, 1246–1258. doi: 10.1111/nph.19225, PMID: 37668195

[ref50] Zheng H. Zhang P. Qin J. Guo J. Deng J. (2023). High-throughput sequencing-based analysis of the composition and diversity of endophytic bacteria community in tubers of Gastrodia elata f.glauca. Front. Microbiol. 13:2552. doi: 10.3389/fmicb.2022.1092552, PMID: 36733772 PMC9887035

[ref51] Zhou M. Wang J. Yang R. Xu X. Lian D. Xu Y.. (2025). Stenotrophomonas sp. SI-NJAU-1 and its mutant strain with excretion-ammonium capability promote plant growth through biological nitrogen fixation. J. Agric. Food Chem. 73, 3874–3886. doi: 10.1021/acs.jafc.4c08697, PMID: 39789791

