# Supplementary figures and images for: Analysis of endophytic bacterial diversity of Puerariae thomsonii from different production areas and their correlation with secondary metabolites
Source: Front Microbiol. 2025 May 26;16:1534308. doi: 10.3389/fmicb.2025.1534308 (PMC12146358; doi:10.3389/fmicb.2025.1534308)

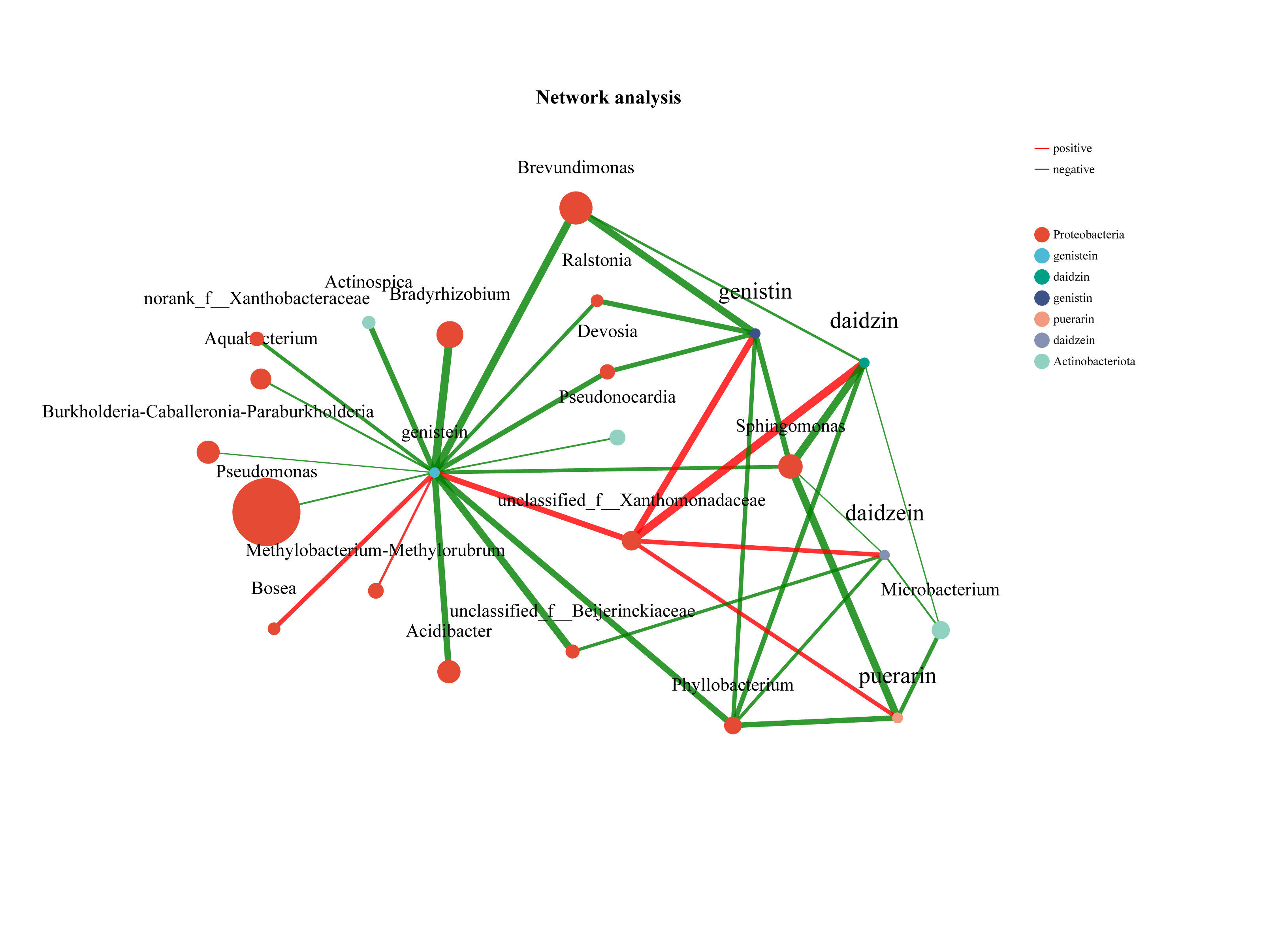

Supplement: SUPPLEMENTARY FIGURE 1 — Association network of endophytic bacteria and metabolites in P. thomsonii samples. Genera with top 30 relative abundance were selected. Pearson correlation coefficients (|r| >0.3; p < 0.05) between bacterial genera and metabolites were calculated. The size of the node in the network is related to the connective degree of the point; the color represents the phylogenetic phyla for bacteria and chemical classes for metabolites; the red and green lines represent the positive and negative correlations between the two nodes, respectively; the thicker the line, the stronger is the correlation. [file Image_1.TIF]

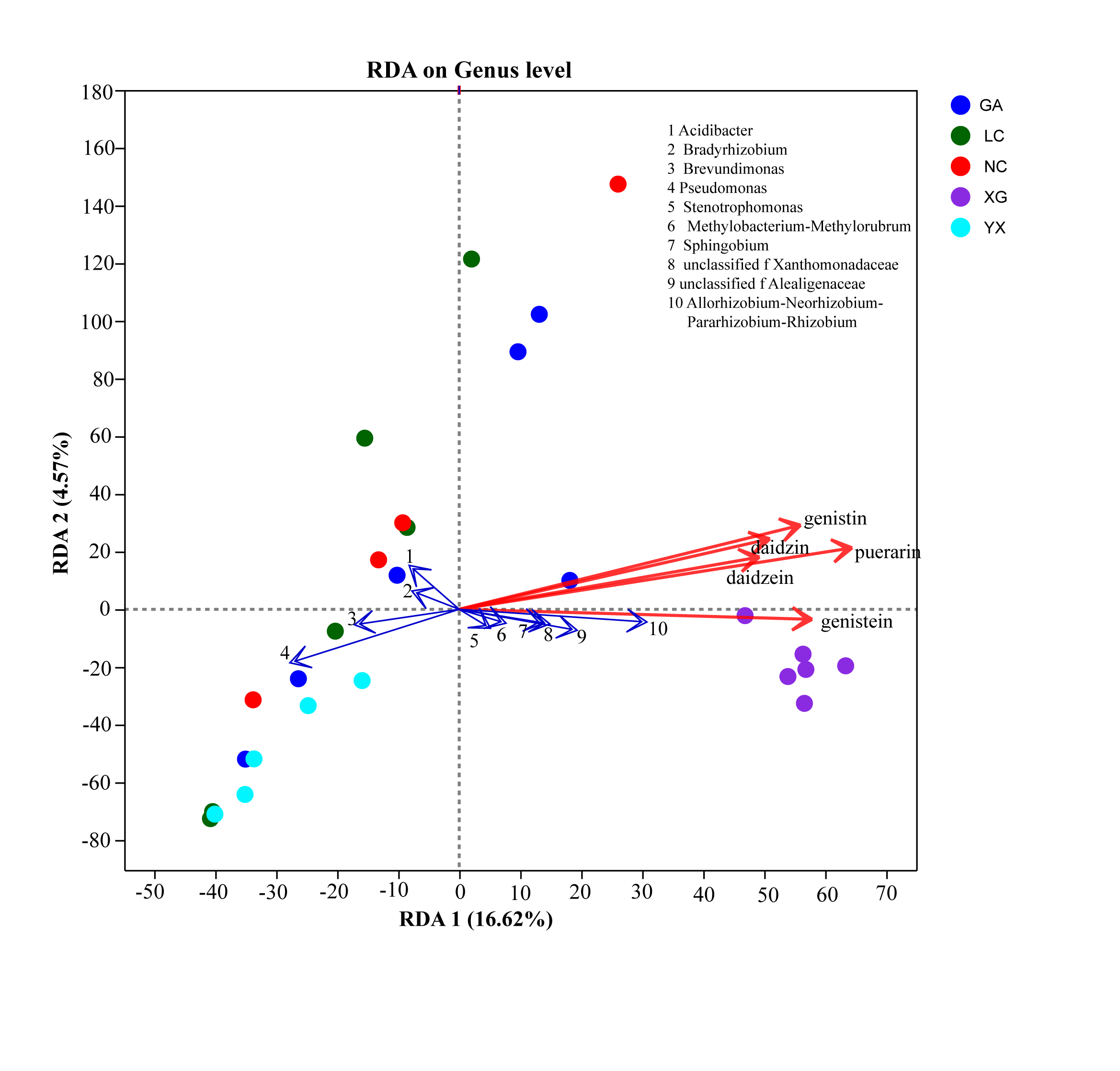

Supplement: SUPPLEMENTARY FIGURE 2 — Redundancy analysis (RDA) of dominant microorganisms and metabolites in P. thomsonii samples. The red arrows represent different metabolites, and the blue arrows represent dominant microorganisms. Circles in different color represent samples from GA, LC, NC, XG, and YX region, respectively. [file Image_2.TIF]
